# The impact of subclinical congestion on the outcome of patients undergoing transcatheter aortic valve implantation

**DOI:** 10.1111/eci.13251

**Published:** 2020-05-15

**Authors:** Christopher Adlbrecht, Felix Piringer, Jon Resar, Victoria Watzal, Martin Andreas, Andreas Strouhal, Waseem Hasan, Daniela Geisler, Gabriel Weiss, Martin Grabenwöger, Georg Delle‐Karth, Markus Mach

**Affiliations:** ^1^ Vienna North Hospital – Clinic Floridsdorf and the Karl Landsteiner Institute for Cardiovascular and Critical Care Research Vienna Vienna Austria; ^2^ Division of Cardiology Department of Medicine Johns Hopkins University School of Medicine Baltimore MD USA; ^3^ Department of Cardio‐Vascular Surgery Hospital Hietzing and Karl Landsteiner Institute for Cardio‐Vascular Research Vienna Austria; ^4^ General Hospital Vienna, Division of Cardiac Surgery Medical University of Vienna Vienna Austria; ^5^ Faculty of Medicine Imperial College London London UK; ^6^ Faculty of Medicine Sigmund Freud University Vienna Austria

**Keywords:** cardiac decompensation, congestion, plasma volume, TAVR, transcatheter aortic valve implantation

## Abstract

**Background:**

We investigated the impact of an elevated plasma volume status (PVS) in patients undergoing TAVI on early clinical safety and mortality and assessed the prognostic utility of PVS for outcome prediction.

**Materials and methods:**

We retrospectively calculated the PVS in 652 patients undergoing TAVI between 2009 and 2018 at two centres. They were then categorized into two groups depending on their preoperative PVS (PVS ≤−4; n = 257 vs PVS>−4; n = 379). Relative PVS was derived by subtracting calculated ideal (iPVS = c × weight) from actual plasma volume (aPVS = (1 − haematocrit) × (a + (b × weight in kg)).

**Results:**

The need for renal replacement therapy (1 (0.4%) vs 17 (4.5%); *P* = .001), re‐operation for noncardiac reasons (9 (3.5%) vs 32 (8.4%); *P* = .003), re‐operation for bleeding (9 (3.5%) vs 27 (7.1%); *P* = .037) and major bleeding (14 (5.4%) vs 37 (9.8%); *P* = .033) were significantly higher in patients with a PVS>−4. The composite 30‐day early safety endpoint (234 (91.1%) vs 314 (82.8%); *P* = .002) confirms that an increased preoperative PVS is associated with a worse overall outcome after TAVI.

**Conclusions:**

An elevated PVS (>−4) as a marker for congestion is associated with significantly worse outcome after TAVI and therefore should be incorporated in preprocedural risk stratification.

## 1 INTRODUCTION

Chronic heart failure (CHF) has a significant impact on the outcome after transcatheter aortic valve implantation (TAVI), yet is widely underestimated in the daily clinical practice.^1^ Since contemporary risk models are based exclusively on left ventricular pump function, newly developed CHF scores represent a decisive decision‐making aid in the preoperative risk assessment.

CHF is a syndrome of a very heterogeneous group of patients with cardiac pathologies. A common feature in all patients with CHF is the extremely poor long‐term prognosis, with mortality curves found to be steeper than in some patients harbouring malignant diseases.^2^

Individuals undergoing TAVI procedure are at great risk of adverse procedural events related to heart failure.^3^ The reason for this is mainly based on two factors: patient selection and the underlying disease. TAVI patients are in general older and suffer from more comorbidities than patients suitable for conventional surgical aortic valve replacement (SAVR).^4^ Aortic stenosis (AS) also inherently leads to left ventricular hypertrophy via increased filling pressures, which ultimately results in heart failure.^5^

Refractory volume overload and congestion, often accelerated by impaired renal function, is one of the biggest concerns in the progression of CHF.^6^ An increase of plasma volume in patients with CHF can lead to acute decompensated heart failure (ADHF) with known adverse effects on prognosis.^2^, ^7^ A recently published score to calculate plasma volume status (PVS) was able to show a correlation between elevated PVS and cardiovascular mortality in patients with stable CHF and PVS greater than −4. In this study, PVS was calculated using only haematocrit, body weight and gender, information easily available for all patients.^8^

Every invasive procedure can trigger ADHF and thereby increase the periprocedural mortality.^9^ As a new tool for risk stratification, calculated PVS was able to show significant results in patients with CHF and patients undergoing coronary bypass graft surgery.^10^

The goal of this study was to determine the impact of preprocedural (often subclinical) cardiac decompensation on early mortality and clinical safety in patients undergoing a TAVI procedure and to assess the predictive power of PVS as a prognostic parameter in preprocedural risk assessment.

## 2 MATERIALS AND METHODS

The study investigated 652 patients who underwent TAVI either via transfemoral (n = 365), transapical (n = 266) or via alternative access (n = 5). Complete data from 532 consecutive patients from the prospectively maintained VICTORY treated between June 2009 and December 2018 at the Heart Center Hietzing/Vienna as well as from 120 consecutive patients of the TAVI Registry from the Johns Hopkins Hospital treated between January and August 2018. The preprocedural assessment and the procedure were performed in a standard fashion by a multidisciplinary heart team and have been previously described in detail.^11^, ^12^ The patient selection process followed the same principles and guidelines at both institutions. As the procedural steps are standardized, comparability of the Austrian and American patient collective can be assumed.

A total of 636 patients had both their weight measured and a complete preprocedural haematologic workup done one day prior to the procedure, and PVS was calculated accordingly. Sixteen patients had to be excluded due to their haematocrit levels having been measured in an extramural setting before the admission for TAVI.

All patients included were educated about the procedure and the associated risks and gave written informed consent. Following approval of the study by the local ethics committees, a retrospective analysis of the patient's baseline characteristics, as well as clinical and procedural data were carried out. Long‐term mortality data including the cause of death were obtained by examination of hospital records and by inquiry to the Federal Institute for Statistics Austria.

Patients were diagnosed with (subclinical) cardiac decompensation when their PVS exceeded threshold levels greater than −4. This cut‐off value derived from the Valsartan in Heart Failure Trial (Val‐HeFT) has proven to be associated with death‐ and morbidity‐related events in its 5248 patient strong analysis.^8^

### 2.1 Plasma volume equations

An equation derived from curve‐fitting techniques using the patients’ haematocrit (Hct) and weight compared to scores of measurements taken from radioisotope assays has been used to calculate the actual plasma volume^13^:

$$\text{actual}\;\text{PV}=\left(\left[1-\text{Hct}\right]\times [a+(b\times \text{weight}[\text{kg}])]\right)$$

Two constants are included in the equation to account for gender differences: *a* = 1530 in males and 864 in females; *b* = 41 in males and 47.9 in females.

The ideal plasma volume was calculated using the following formula described by Longo et al^14^

idealPV=c×weight(kg)

where *c* is a constant accounting for gender differences, equivalent to 39 in males and 40 in females.

Subsequently, the relative plasma volume describing the patients percentual deviation from their ideal plasma volume was calculated using the following Equation ^8^:

$$\text{PVS}=\left(\left[\text{actual}\;\text{PV}-\text{ideal}\;\text{PV}\right]/\text{ideal}\;\text{PV}\right)\times 100\%$$

The clinical outcome and the occurrence of related peri‐ and postprocedural complications were classified as per the updated Valve Academic Research Consortium (VARC)‐II criteria.^15^ The primary study endpoint was defined as 30‐day mortality; the composite secondary endpoint was defined as early safety at 30‐days incorporating the freedom of all‐cause mortality, stroke, life‐threatening bleeding, acute kidney injury stage 2 or 3, coronary artery obstruction requiring intervention, major vascular complication and valve‐related dysfunction requiring repeat procedure. Long‐term survival was assessed between the two groups.

### 2.2 Statistical analysis

The study population was separated into two cohorts: those who presented with a relative PVS > −4 prior to TAVI and those who had a relative PVS score ≤−4. Continuous variables were—based on their distribution—expressed as either a median and interquartile range (IQR) or a mean and standard deviation (±SD) and compared using the Student's *t* test or the Mann‐Whitney *U* test, respectively. Categorical variables were expressed as absolute numbers and percentage and compared with a Chi^2^ test or the Fisher's exact test.

To examine the association between the PVS and the overall long‐term mortality, a Cox proportional hazards model was used to estimate hazard ratios and 95% confidence intervals. The date of the implantation to either death or the last available follow‐up was used to calculate the individual person‐time interval. The hazard ratio was stratified by the PVS score and adjusted for baseline characteristics including the STS score, and both the logistic EuroSCORE and the EuroSCORE II in a stepwise fashion.

All reported p‐values are two‐sided, and results were categorized as statistically significant with an alpha level set at <0.05; the analyses were performed using spss, version 24.0 (IBM Corp).

## 3 RESULTS

In total, the study investigated 636 patients. The preprocedural medical history and the patients’ risk profile are outlined in Table 1. According to the PVS, almost two‐thirds of all patients referred for TAVI were in (subclinical) cardiac decompensation (PVS > −4: n = 379; 59.6%). While the demographic data of the overall study cohort were representative of the current population treated with TAVI (mean age 80.2 ± 7.4 years, female n = 389 [61.2%], mean BMI 26.1 ± 6.7), there was a significant difference in age between the two study cohorts.^16^, ^17^ Patients with a PVS > −4 were generally older (80.8 ± 7.2 years vs 79.3 ± 7.6 years; *P* = .009), the percentage of female patients was higher (250 [66%] vs 139 [54.1%]; *P* = .002) and the BMI was significantly lower (25.3 ± 5.7 vs 29.4 ± 5.3; *P* < .001). Given that patients in the higher PVS cohort had a greater plasma volume, the lower BMI seems indicative of a generally worse health status or frailty among these patients. Accordingly, the EuroSCORE II (5 ± 5.1 vs 3.8 ± 3.9; *P* < .001) and the STS score (4.9 ± 3.8 vs 3.7 ± 2.7; *P* < .001) were significantly higher for patients in the higher PVS cohort. Furthermore, patients with a higher PVS were more symptomatic (NYHA III/IV: 313 (82.6%) vs 194 (75.5%); *P* = .019), significantly more often oxygen dependent (10 (2.6%) vs 1 (0.4%); *P* = .028) but suffered less often from obstructive sleep apnoea (OSAS) (10 (2.6%) vs 21 (8.2%); *P* = .001). It is interesting to note that there was no apparent difference in the preprocedural serum creatinine level among the groups (1.1 (0.7) vs 1.1 (0.5); *P* = .328). However, there were more patients with a glomerular filtration rate below 30ml/min (20.6% vs 12.8%; *P* = .006) in those classified decompensated by higher PVS.

**Table 1:** Baseline clinical characteristics

	Overall n = 636		PVS ≤ −4 n = 257		PVS> −4 n = 379		*P* value
Demographics							
Age, mean (±SD)	80.2	(7.4)	79.3	(7.6)	80.8	(7.2)	**.009**
Female, n (%)	389	(61.2)	139	(54.1)	250	(66.0)	**.002**
Body mass index kg/m^2^, mean (±SD)	26.1	(6.7)	29.4	(5.3)	25.3	(5.7)	**<.001**
Risk profile							
Logistic EuroSCORE, median (±IQR)	15.1	(14.1)	13.6	(13.4)	16.2	(16.0)	**.009**
EuroSCORE II, median (±IQR)	4.4	(4.8)	3.8	(3.9)	5	(5.1)	**<.001**
STS score, median (±IQR)	4.5	(3.3)	3.7	(2.7)	4.9	(3.8)	**<.001**
Chronic health conditions and risk factors							
Dyslipidaemia, n (%)	412	(64.8)	174	(67.7)	238	(62.8)	.126
Diabetes mellitus (IDDM), n (%)	127	(20.0)	54	(21.0)	73	(19.3)	.329
Hypertension, n (%)	564	(88.7)	233	(90.7)	311	(82.1)	.159
COPD, n (%)	76	(11.9)	28	(10.9)	48	(12.7)	.300
Peripheral vascular disease, n (%)	120	(18.9)	40	(15.6)	80	(21.1)	**.047**
Creatinine mg/dL, median (IQR)	1.1	(0.6)	1.1	(0.5)	1.1	(0.7)	.328
Renal impairment eGFR < 30 mL/min/1.73 m^2^, n (%)	111	(17.5)	33	(12.8)	78	(20.6)	**.006**
Dialysis, n (%)	4	(0.6)	2	(0.8)	2	(0.5)	.485
Cerebrovascular accident, n (%)	80	(12.6)	37	(14.4)	43	(11.3)	.158
Preprocedural atrial fibrillation, n (%)	191	(30.0)	79	(30.7)	112	(29.6)	.412
Oxygen dependence, n (%)	11	(1.7)	1	(0.4)	10	(2.6)	**.028**
Obstructive sleep apnoea, n (%)	31	(4.9)	21	(8.2)	10	(2.6)	**.001**
NYHA III/IV, n (%)	507	(79.7)	194	(75.5)	313	(82.6)	**.019**
Coronary vascular disease present, n (%)	335	(52.7)	138	(53.7)	197	(52.0)	.375
Prior myocardial infarction, n (%)	107	(16.8)	36	(14.0)	71	(18.7)	.072
Previous PCI, n (%)	202	(31.8)	84	(32.7)	118	(31.1)	.380
Previous pacemaker implantation, n (%)	94	(14.8)	37	(14.4)	57	(15.0)	.458
Previous CABG, n (%)	99	(15.6)	44	(17.1)	55	(14.5)	.217
Previous valve surgery, n (%)	59	(9.3)	25	(9.7)	34	(9.0)	.429
Preoperative echocardiographic data							
Aortic valve area, mean (±SD)	0.68	(0.2)	0.73	(0.3)	0.68	(0.3)	**.001**
Indexed aortic valve area, mean (±SD)	0.36	(0.1)	0.35	(0.2)	0.36	(0.2)	**.146**
Mean pressure gradient, mean (±SD)	45.6	(17.2)	44.3	(16.1)	46.5	(17.8)	.111
Max. pressure gradient, mean (±SD)	71.3	(22.0)	69.1	(20.4)	72.8	(22.8)	.061
Peak velocity m/sec, median (±IQR)	4.1	(0.9)	4.1	(0.8)	4.2	(1.0)	.248
sPAP, mean (±SD)	37.1	(23.6)	33.7	(23.5)	39.1	(23.6)	**.018**
LVEF %, median (±IQR)	55	(15.0)	55	(15.0)	55	(15.0)	.376
Low‐flow/low‐gradient stenosis, n (%)	94	(27.0)	40	(30.3)	54	(25.0)	.346

Abbreviations: CABG, coronary artery bypass graft; CHA2DS2‐VASc, Congestive heart failure, Hypertension, Age > 75 y, Diabetes mellitus, Stroke or embolic event, Vascular disease, Age 65‐74 y, Sex; COPD, chronic obstructive pulmonary disease; eGFR, estimated glomerular filtration rate; EuroSCORE, European System for Cardiac Operative Risk Evaluation; HAS‐BLED, Hypertension, Abnormal renal or liver function, Elderly, Stroke, prior major Bleeding or predisposition, Labile INR, Drugs; IDDM, insulin‐dependent diabetes mellitus; IQR, interquartile range; LVEF, left ventricular ejection fraction; Max., maximum; NYHA, New York Heart Association; other abbreviations as in Table 1; PCI, percutaneous coronary intervention; SD, standard deviation; sPAP, systolic pulmonary artery pressure; STS, Society of Thoracic Surgeons Predictive Risk of Mortality.

*P* values indicating significant differences between cohorts are highlighted in bold

The preoperative echocardiographic investigation demonstrated that patients with a PVS higher than −4 had a significantly higher systolic pulmonary artery pressure (39.1 ± 23.6 vs 33.7 ± 23.5 mm Hg; *P* = .018). Importantly, no significant difference in preoperative left ventricular ejection fraction was observed between the two PVS cohorts.

### 3.1 Procedural and postinterventional characteristics

The procedural and postinterventional characteristics are outlined in Table 2. In our study, patients with a higher PVS were generally smaller in stature and consequently also received smaller sized prosthetic valves (27 ± 2.9 mm vs 26.3 ± 2.6 mm; *P* = .007). The significantly increased requirement for predilatation (53.3% vs 64.9%; *P* = .002) and reduced amount of radiation exposure (9794 cGy vs 7199 cGy; *P* < .001) may be indicative of a higher calcific burden in these patients. Furthermore, patients with a higher PVS score demonstrated a longer stay at both the intensive care unit and the general ward (20 ± 44 hours vs 21 ± 50 hours; *P* = .001 and 8 ± 11 vs 10 ± 9 days; *P* = .001, respectively) and showed a strong trend towards prolonged postprocedural ventilation (0 ± 6.0 hours vs 2.5 ± 6.0 hours; *P* = .053).

**Table 2:** Procedural clinical characteristics

	Overall n = 636		PVS ≤ −4 n = 257		PVS > −4 n = 379		*P* value
Procedural variables							
Skin‐to‐skin time (min), median(±IQR)	87	(31.0)	88	(32.0)	85	(31.0)	.202
Balloon expanding valve, n (%)	294	(46.2)	119	(46.3)	175	(46.2)	.519
Prosthesis size (mm), mean (±SD)	26.6	(2.7)	27	(2.9)	26.3	(2.6)	**.007**
Predilatation, n (%)	383	(60.2)	137	(53.3)	246	(64.9)	**.002**
Postdilatation, n (%)	101	(15.9)	38	(14.8)	63	(16.6)	.306
Paravalvular leak (moderate/severe), n (%)	8	(1.3)	5	(1.9)	3	(0.8)	.181
Fluoro time (min), median (IQR)	15.2	(10.0)	15.4	(8.5)	14.6	(10.5)	.071
Absorbed radiation cGy, median (±IQR)	7925	(13 411)	9794	(33 312)	7199	(10 044)	**<.001**
Contrast volume (cc), median (±IQR)	150	(141.5)	148	(148.0)	150	(140.5)	.156
Total hours in ICU, median (±IQR)	20	(48.0)	20	(44.0)	21	(50.0)	**.001**
Total hours ventilated, median (±IQR)	0	(6.0)	0	(6.0)	2.5	(6.0)	.053
RBC units used, mean (±SD)	1.2	(2.7)	0.4	(1.2)	1.6	(3.2)	**<.001**
Max. creatinine within 72 h mg/dL, median (±IQR)	1.1	(0.6)	1	(0.5)	1.1	(0.7)	.449
Mean gradient postimplant, median (±IQR)	9	(7.0)	9.3	(8.0)	9	(6.0)	.203
Max. gradient postimplant median (±IQR)	17.5	(13.0)	18	(13.0)	17.2	(12.0)	.367
Max. flow postimplant, median (±IQR)	2	(1.0)	2.1	(1.0)	2	(1.0)	.129
Length of stay after TAVI (d), median (±IQR)	9	(8.0)	8	(11.0)	10	(9.0)	**.001**

Abbreviations: BMI, body mass index; BW, body weight; CM, contrast medium; ICU, intensive care unit; other abbreviations as in Tables 1 and 2; RBU, red blood cell.

*P* values indicating significant differences between cohorts are highlighted in bold

### 3.2 Adverse events and survival

Significantly more patients in the PVS > −4 cohort were in need of postoperative renal replacement therapy (0.4% vs 4.5%; *P* = .001) and of reoperation/reintervention for noncardiac reasons (3.5% vs 9.2%; *P* = .003). The latter comprise mostly the need for pleural drainage placement. Moreover, patients with an elevated PVS score were more prone to major bleeding events (5.4% vs 9.8%; *P* = .033) resulting in an increased requirement for red blood cell transfusions (0.4 vs 1.6 units; *P* = .001).

Although no difference in 30‐day all‐cause mortality (3.5% vs 5.8%; *P* = .127) was demonstrated between the two groups, patients with a higher PVS at baseline reached the procedural safety endpoint at 30‐days less frequently (82.3% vs 88.9%; *P* = .042), thus confirming the notion that an elevated PVS increases the overall risk for patients undergoing TAVI procedure (Table 3).

**Table 3:** Adverse events

	Overall n = 636		PVS ≤ −4 n = 257		PVS > −4 n = 379		*P* value
Adverse events data							
Myocardial infarction, n (%)	4	(0.6)	0	(0.0)	4	(1.1)	.255
Major vascular access complication, n (%)	17	(2.7)	6	(2.3)	11	(2.9)	.683
Major bleeding complication, n (%)	51	(8.0)	14	(5.4)	37	(9.8)	**.033**
Neurological adverse event, n (%)	15	(2.4)	4	(1.6)	11	(2.9)	.205
Acute kidney injury, n (%)	84	(13.2)	25	(9.7)	59	(15.6)	.065
Postoperative renal replacement therapy, n (%)	18	(2.8)	1	(0.4)	17	(4.5)	**.001**
New atrial fibrillation, n (%)	54	(8.5)	18	(7.0)	36	(9.5)	.302
New pacemaker implanted, n (%)	82	(12.9)	32	(12.5)	50	(13.2)	.441
Pneumonia under antibiotic treatment, n (%)	33	(5.2)	12	(4.7)	21	(5.5)	.386
Conversion to open surgery, n (%)	9	(1.4)	4	(1.6)	5	(1.3)	.528
Reoperation for valvular dysfunction, n (%)	4	(0.6)	2	(0.8)	2	(0.5)	.532
Reoperation for bleeding/tamponade, n (%)	36	(5.7)	9	(3.5)	27	(7.1)	**.037**
Reoperation for other cardiac problems, n (%)	48	(7.5)	16	(6.2)	32	(8.4)	.188
Reoperation for noncardiac problems, n (%)	44	(6.9)	9	(3.5)	35	(9.2)	**.003**
Operational death, n (%)	10	(1.6)	5	(1.9)	5	(1.3)	.376
30‐d composite early safety endpoint, n (%)	548	(86.2)	234	(91.1)	314	(82.8)	**.002**
30‐d all‐cause mortality, n (%)	31	(4.9)	9	(3.5)	22	(5.8)	.127

Abbreviation: AV, atrioventricular; other abbreviations as in Tables 1, 2, 3.

*P* values indicating significant differences between cohorts are highlighted in bold

**Figure 1:**
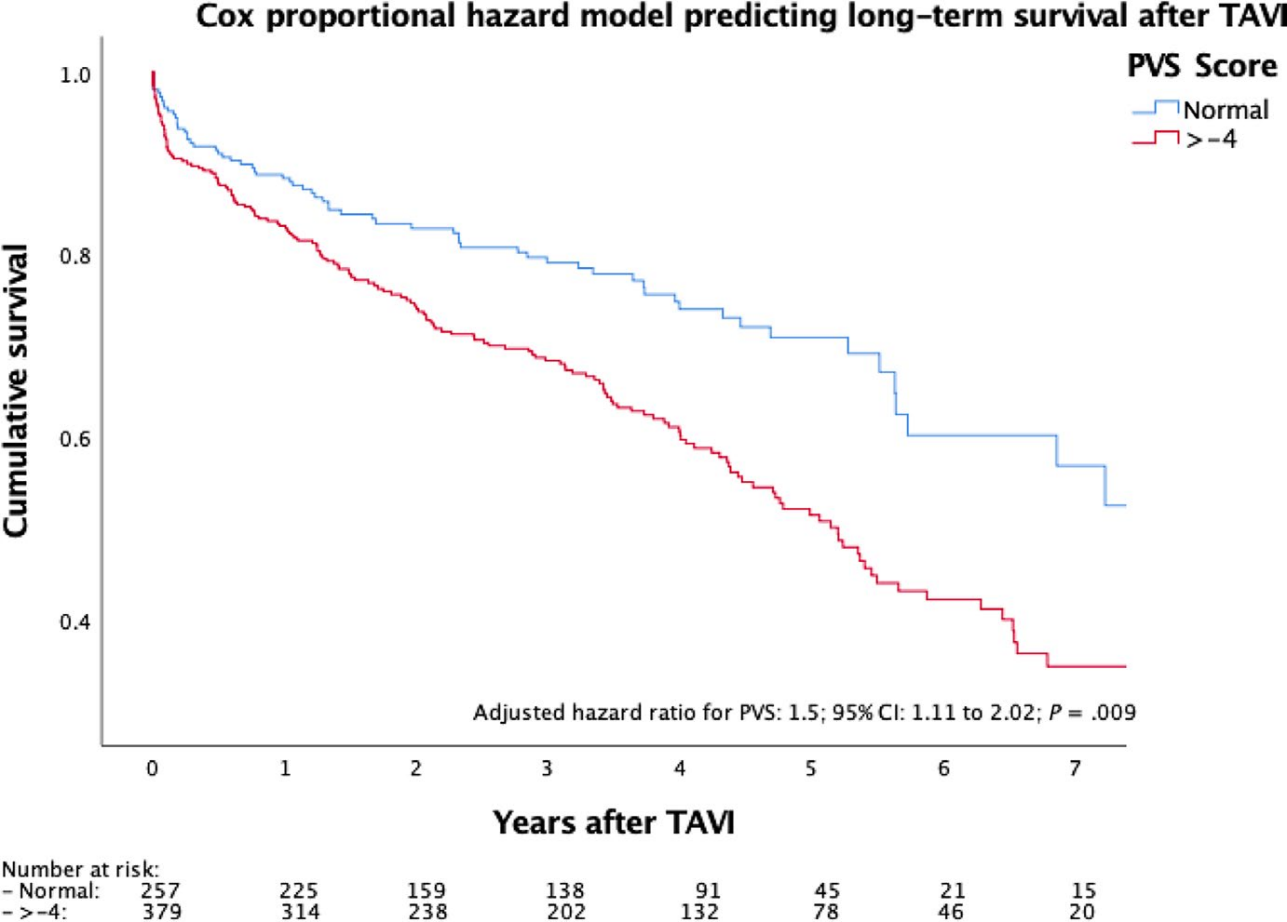
Cox regression model predicting long‐term survival after TAVI of patients with low versus high PVS; *Legend: Survival function of means for patients with low versus high PVS after TAVI*

Adjusting the Cox proportional hazards model for the STS score, as well as the EuroSCORE II, the treatment centre, the access site and the investigation period tertiles (2009‐2011; 2012‐2014; 2015‐2018), a significantly lower long‐term survival of patients with a higher PVS was demonstrated (adjusted hazard ratio: 1.5; 95% CI: 1.11‐2.02; *P* = .009; Figure 1).

## 4 DISCUSSION

We assessed the association of (sub‐) clinical decompensation determined by PVS and outcomes in patients with severe, symptomatic AS undergoing TAVI.

Every TAVI procedure has an impending risk for adverse events related to heart failure.^3^ Patients in need of TAVI are frequently at advanced age with symptomatic AS, and multiple preexisting conditions and comorbidities. Generally, in hospital admissions for ADHF, increased congestion is associated with morbidity and mortality.^7^ In line with these findings, our data show that cardiac decompensation defined by the PVS status was associated with worse short‐ and long‐term outcomes. The potential implications of this become evident when considering that about two‐thirds (59.6%) of the patients in our study admitted for TAVI were in cardiac decompensation. These patients were not necessarily clinically decompensated but defined by an elevated PVS rather subclinically.

These patients had a significantly lower BMI presumably underlining the overall impaired health and advanced disease progression of this population. Taking comorbidities into account, iron deficiency represents a plausible pathophysiological link, as decongestion according to the PVS could be achieved by intravenous iron repletion in CHF patients with iron deficiency.^18^ Considering anaemia is a well‐established predictor unfavourable outcome in CHF, iron deficiency along with several other factors such as haemodilution may be involved in the underlying mechanism.^19^

In the present study, we were able to show that the calculated PVS may help identify patients at risk for adverse outcomes. Patients with a high PVS were not only at increased risk for periprocedural death but also demonstrated a substantially impaired long‐term survival during the follow‐up period. This association remained statistically significant even after adjusting for routinely applied clinical risk scores, such as the EuroSCORE II.

There is a plethora of causes for CHF including ischaemia, amyloidosis, toxins, arrhythmia and notably in the context of this paper, structural pathologies. Similarly, the potential reasons or triggers for cardiac decompensation are manifold as well.^20^ In the setting of severe AS, it seems that impaired left ventricular function as the main cause of CHF is of minor importance, as the leading mechanism may be either the mechanical flow impairment alone or in combination with a low‐flow state due to impaired ventricular ejection and/or diastolic dysfunction.

Our study shows that calculated PVS exhibited predictive power in TAVI patients with respect to our 30‐day composite safety endpoint, and was associated with a significantly worse long‐term prognosis in patients with PVS > −4. However, the shown data raise several questions and further research in this area is warranted. Potential optimizations of pre‐ or postoperative management have to be evaluated. This may include adaptation of diuretic therapy, intravenous iron repletion and heart failure‐specific medical therapy. In addition to that, PVS calculation is particularly useful in timing of the TAVI procedure by identifying the temporal “sweet spot” of complete recompensation. The PVS could represent an objective parameter for the identification of patients who qualify for a “fast track” implantation slot. Moreover, differences in PVS may influence the local timing of patients when several TAVI procedures are performed on the same day, meaning that identification of the patient at highest risk for complications could be scheduled to an intensive care unit postinterventionally and those at lower risk probably qualify for an intermediate care or general care unit. Reviewing the results of this study, PVS calculation might answer decisive questions in the therapy of asymptomatic patients with aortic valve stenosis. In‐depth assessment of changes underlying increased PVS may include extracellular matrix increase/myocardial fibrosis, amyloidosis and many others.

### 4.1 Limitations

The study is limited by its retrospective design and could have been subject to a selection bias as patients with more severe, clinically manifest decompensation may have been admitted to hospital before the procedure and then recompensated. Consequently, the reported proportion of decompensated patients may actually be even higher if these patients were included into the calculation. Additionally, we were unable to correlate PVS calculations to measurements of natriuretic peptides as these were unavailable for a significant number of the study population. Certain technical advances and the general increase of operator experience over time, as well as the inclusion of different access pathways and study centres may have added to the heterogeneity of the study population.

## 5 CONCLUSION

Even though not directly affecting the early mortality after TAVI, the PVS represents a useful tool for clinicians to assess the risk for adverse outcomes in patients undergoing a TAVI procedure. Patients presenting with a higher PVS score at the time of admission for TAVI displayed an increased risk for postprocedural renal replacement therapy, bleeding complications, the need of reoperation for noncardiac reasons and impaired long‐term survival. The fact that most patients in our population had a preserved left ventricular ejection fraction (LVEF) suggests that mechanical obstruction is a more relevant precipitator of decompensation than low LVEF in patients with severe AS. Calculated PVS may be used to prioritize patients waiting for a TAVI and may simultaneously represent a marker for futile treatment in TAVI candidates.

### 5.1 Impact on daily practice

The study demonstrates the importance for patients receiving optimized medical therapy for complete cardiac recompensation prior to TAVI. Furthermore, the calculation of patient's plasma volume status is an effective method that aids preprocedural risk stratification and timing of the procedure.

## CONFLICT OF INTEREST

Dr Mach has received a research grants from Edwards Lifesciences, JenaValve and Symetis. Dr. Andreas is a proctor for Edwards Lifesciences and Abbott Medical and an advisor for Medtronic. All other authors have reported that they have no relationships relevant to the content.
